# Cloning and expression of *R-Spondin1 *in different vertebrates suggests a conserved role in ovarian development

**DOI:** 10.1186/1471-213X-8-72

**Published:** 2008-07-24

**Authors:** Craig A Smith, Christina M Shoemaker, Kelly N Roeszler, Joanna Queen, David Crews, Andrew H Sinclair

**Affiliations:** 1 The University of Melbourne Department of Paediatrics and Murdoch Childrens Research Institute, Royal Children's Hospital, Melbourne, Victoria, 3052, Australia; 2 Section of Integrative Biology, University of Texas, Austin, Texas, USA

## Abstract

**Background:**

R-Spondin1 (Rspo1) is a novel regulator of the Wnt/β-catenin signalling pathway. Loss-of-function mutations in human *RSPO1 *cause testicular differentiation in 46, XX females, pointing to a role in ovarian development. Here we report the cloning and comparative expression analysis of *R-SPONDIN1 *orthologues in the mouse, chicken and red-eared slider turtle, three species with different sex-determining mechanisms. Evidence is presented that this gene is an ancient component of the vertebrate ovary-determining pathway.

**Results:**

Gonadal *RSPO1 *gene expression is female up-regulated in the embryonic gonads in each species at the onset of sexual differentiation. In the mouse gonad, *Rspo1 *mRNA is expressed in the somatic cell lineage at the time of ovarian differentiation (E12.5–E15.5), with little expression in germ cells. However, the protein is localised in the cytoplasm and at the cell surface of both somatic (pre-follicular) and germ cells. In the chicken embryo, *RSPO1 *expression becomes elevated in females at the time of ovarian differentiation, coinciding with female-specific activation of the *FOXL2 *gene and estrogen synthesis. RSPO1 protein in chicken is localised in the outer cortical zone of the developing ovary, the site of primordial follicle formation and germ cell differentiation. Inhibition of estrogen synthesis with a specific aromatase inhibitor results in a decline in chicken *RSPO1 *expression, indicating that RSPO1 is influenced by estrogen. In the red-eared slider turtle, which exhibits temperature-dependent sex determination, up-regulation of *RSPO1 *occurs during the temperature-sensitive period, when gonadal development is responsive to temperature. Accordingly, *RSPO1 *expression is temperature-responsive, and is down-regulated in embryos shifted from female- to male-producing incubation temperatures.

**Conclusion:**

These results indicate that *RSPO1 *is up-regulated in the embryonic gonads of female vertebrates with different sex-determining mechanisms. In all instances, *RSPO1 *is expressed in the incipient ovary. These findings suggest that *R-SPONDIN1 *is an ancient, conserved part of the vertebrate ovary-determining pathway.

## Background

Sex determination in vertebrate embryos results in either testis or ovary development. In most mammals, the Y-linked *SRY *gene initiates testicular development by directing Sertoli cell differentiation and the organization of seminiferous cords [1-5]. Leydig and other cell types then differentiate around the cords, testosterone is released and male development is elaborated. A number of transcription factors and signalling molecules are now known to play a role in testicular differentiation upstream and downstream of SRY. These include *GATA4, FOG2*, *WT1, SOX9, SF1, DAX1 *and *FGF9 *(reviewed in [6]). In contrast, the molecular control of ovarian development is less well understood. In XX mouse embryos, the earliest signs of ovarian differentiation include entry of primordial germ cells into the first stages of meiosis, followed by a fragmentation of primitive sex cords around the germ cells and their organization into primordial follicles. Expression based screens have shown that the differentiating ovary has a robust program of gene expression, equalling that of the testis [7]. One requirement for proper ovarian development is Wnt signalling. The *Wnt4 *gene is up-regulated in embryonic mouse XX gonads, while loss-of-function mutants have masculinized XX gonads [8-10]. Conversely, testicular vascular development and androgen production are retarded in XY embryos over-expressing *Wnt4 *[10,11]. However, *Wnt4 *has been shown to play a role in both testis and ovary formation, since loss of Wnt4 signalling interferes with proper Sertoli cell development in males [12]. Most recently, it has been shown that compound mutants lacking both *Wnt4 *and the transcription factor *Foxl2 *develop as XX males, with patent testes [13]. These observations point to an important additive role for these two genes in ovarian differentiation.

A new gene implicated in female development is *R-SPONDIN1 *(*RSPO1*). This gene was identified in humans through linkage analysis of a family and an isolated individual with hyperkeratinised skin, a predisposition to squamous cell carcinoma and 46, XX female-to-male sex reversal. Two separate loss of function mutations were found in the *RSPO1 *gene of affected patients: a single nucleotide insertion resulting in a premature stop codon and a 2.5 kb deletion spanning exon 4 [14]. These mutations resulted in testis formation and male development in XX individuals, in the absence of *SRY*. It follows that *RSPO1 *plays a role in ovarian development and that repression of *RSPO1 *is compatible with testicular development. Mouse *Rspo1 *is expressed in those tissues affected by *RSPO1 *mutations in humans, such as the skin and embryonic gonads. Very recently, the mouse *Rspo1 *null mutant has been described. *Rspo1*^-/- ^XX mice are masculinized, with depleted germ cells, male-like vascularisation, deregulation of *Wnt4 *expression and ectopic testosterone production [15,16]. Altogether, these data indicate that *RSPO1 *is an important gene involved in mammalian ovarian development.

RSPO1 is a member of a small family of secreted growth factors. The four *R-spondin *genes in mouse show similar expression patterns to Wnt signalling molecules, and Rspo proteins can operate through the canonical Wnt signalling pathway, stabilising intracellular β-catenin [17-19]. β-catenin can then enter the nucleus, where it interacts with members of the TCF/LEF family of transcription factors to regulate gene expression. The Rspo proteins can therefore potentially regulate functions mediated by β-catenin, such as cell fate decisions and embryonic patterning. Recent data indicate that RSPO1 stimulates the β-catenin signalling pathway by binding the WNT co-receptor, LRP6, modulating its availability [20,21]. It is possible that Rspo proteins may also operate independently of Wnt signalling [22]. However, given the relationship between Rspo proteins and the Wnt pathway, and the importance of Wnt4 for gonadal development, these two factors might interact to play a role in ovary development [16,22].

It has been suggested that *RSPO1 *could represent the key ovary-determining gene in humans, and possibly all mammals [14,22]. However, XX *Rspo1 *null mutant mice are only partially sex-reversed, implying that *Rspo1 *alone is not the key "ovary-determining gene" (at least in mammals). Here, we describe the spatial and temporal expression profiles of *Rspo1 *in the embryonic gonads of the mouse, chicken (*Gallus gallus*) and the red-eared slider turtle (*Trachemys scripta*). These three species have different sex-determining mechanisms; XY male heterogamety in mouse, ZW female heterogamety in chicken and temperature-dependent sex determination (TSD) in the turtle. Despite these different sex-determining triggers, *RSPO1 *shows a conserved ovary-specific expression profile in all three species. These observations suggest that *RSPO1 *has an important role in ovarian development in amniotic vertebrates.

## Results

### *RSPO1 *orthologues

**Figure 1:**
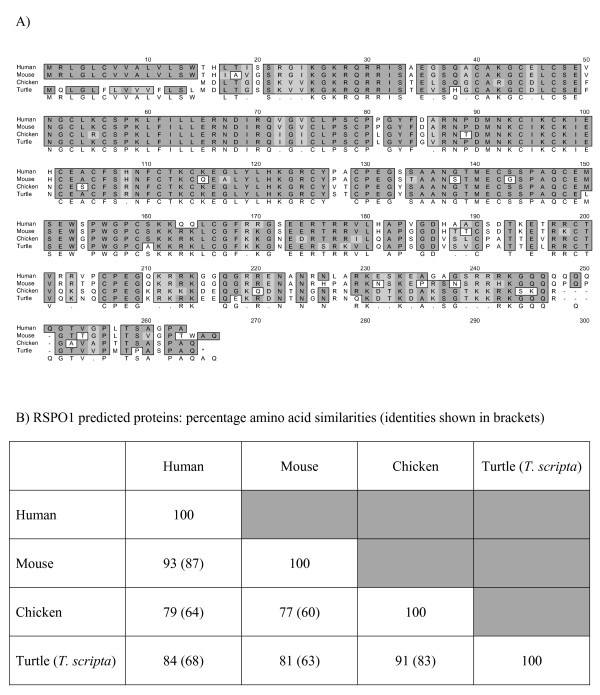
**A. Alignment of predicted R-SPONDIN1 proteins in human, mouse, chicken and turtle (*T. scripta)*.** B. Percentage amino acid similarities (identities) for RSPO1 orthologues.

PCR of gonadal cDNA was used to amplify chicken and turtle (*T. scripta*) orthologues of mammalian *RSPO1*. Figure 1A shows an alignment of the predicted amino acid sequences for human, mouse, chicken and turtle RSPO1. Human and mouse RSPO1 proteins are very similar, with 93% amino acid similarity (87% identity). The predicted sequence derived from the chicken genome database lacks 14 residues at the amino terminal relative to human *RSPO1*. We have cloned the entire open reading *T. scripta RSPO1 *(784 base pair open reading frame; 261 amino acids). Overall, the chicken and human RSPO1 proteins exhibit 79% amino acid similarity. Turtle (*T. scripta*) RSPO1 shares greatest homology with the chicken sequence (91% amino acid similarity), followed by the human (84% similarity) and mouse (81% similarity) (Figure 1B).

### Expression of Rspo1 in sorted mouse gonadal cells

**Figure 2:**
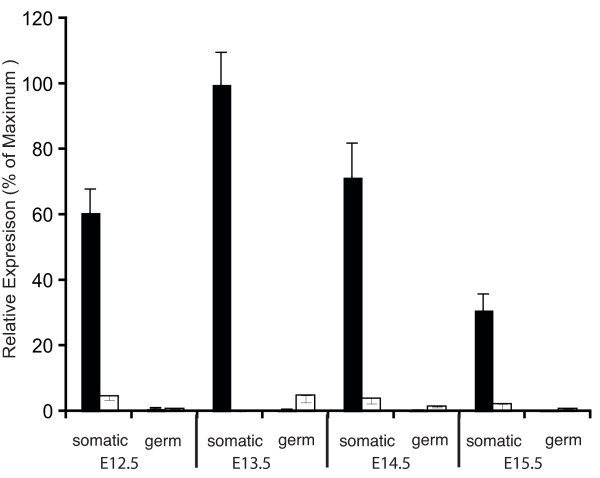
**Expression of mouse *Rspo1 *mRNA in FACS-sorted gonadal somatic and germ cells, over E12.5 – 15.5**. Mean +/- SEM. ■ = female; □ = male.

We firstly examined mouse *Rspo1 *expression in FACS sorted embryonic gonadal somatic and germ cells. Embryonic gonads from OCT4-GFP transgenic mice were dissociated and sorted on the basis of GFP expression (confined to the germline). The sorted cells have been shown to be pure populations of somatic and germ cells [23]. Real time PCR analysis showed that mouse *Rspo1 *was expressed predominantly in the somatic cell compartment, and up-regulated in XX gonads (Fig. 2). In the mouse embryo, the onset of gonadal sex differentiation occurs at E12.5, one day after the peak of *Sry *expression in male (XY) gonads. At the earliest time point examined here, E12.5, *Rspo1 *expression was already strongly sexually dimorphic, being up-regulated in females. Expression in somatic cells of XY embryos was very low through the period of gonadal sex differentiation, from embryonic day (E) 12.5 – 15.5. There was a low level of *Rspo1 *mRNA expression in the germ cells in both sexes, over E12.5 – E15.5.

### Chicken RSPO1 expression and effects of aromatase inhibition

**Figure 3:**
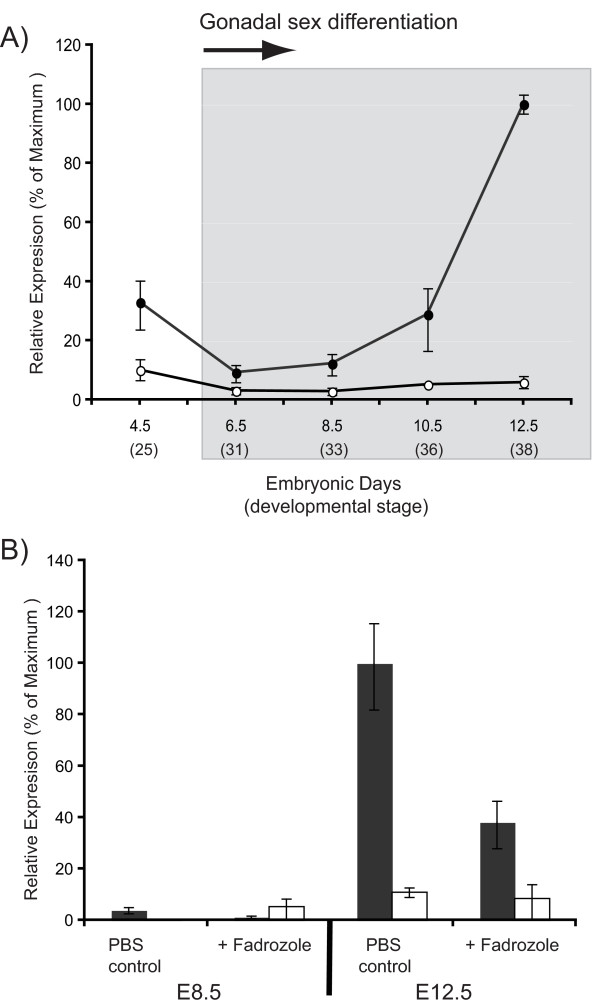
**A) Expression of *RSPO1 *mRNA in embryonic chicken gonads over development (Embryonic days (E) (stages), assayed by qRT-PCR**. Values represent mean +/- SEM. ● = female; ○ = male. B) Effect of fadrozole aromatase inhibitor on gonadal *RSPO1 *expression in the chicken embryo. Embryos were treated at E3.5 with 0.1 ml PBS control or 0.1 ml (1 mg) fadrozole in PBS, and examined at E8.5 and E12.5. Quantitative RT-PCR. Mean +/- SEM. ■ = female; □ = male.

Chicken *RSPO1 *showed sexually dimorphic expression, being up-regulated in the presumptive ovary of ZW embryos from early stages (Fig. 3A). In ZW (genetic female) embryos, gonadal *RSPO1 *expression was elevated as early as E4.5, and became strongly elevated from E8.5. Gonads were not separated from the mesonephric kidneys at E4.5, hence, expression for that time point pertains to the entire urogenital system. Expression in the gonads of ZZ embryos (presumptive males) remained low. In chicken embryos treated with the specific aromatase inhibitor, fadrozole, *RSPO1 *expression declined (Fig. 3B). In these experiments, eggs were treated with aromatase inhibitor or PBS control on day 3.5 and RNA was extracted from gonads on day 8.5 or day 12.5. *RSPO1 *was lowly expressed at E8.5, but was nevertheless inhibited in fadrozole treated embryos compared to PBS treated controls. By E12.5, when *RSPO1 *expression was high in control females and low in males, fadrozole treatment resulted in a dramatic decline in *RSPO1 *in females (Fig. 3B).

**Figure 4:**
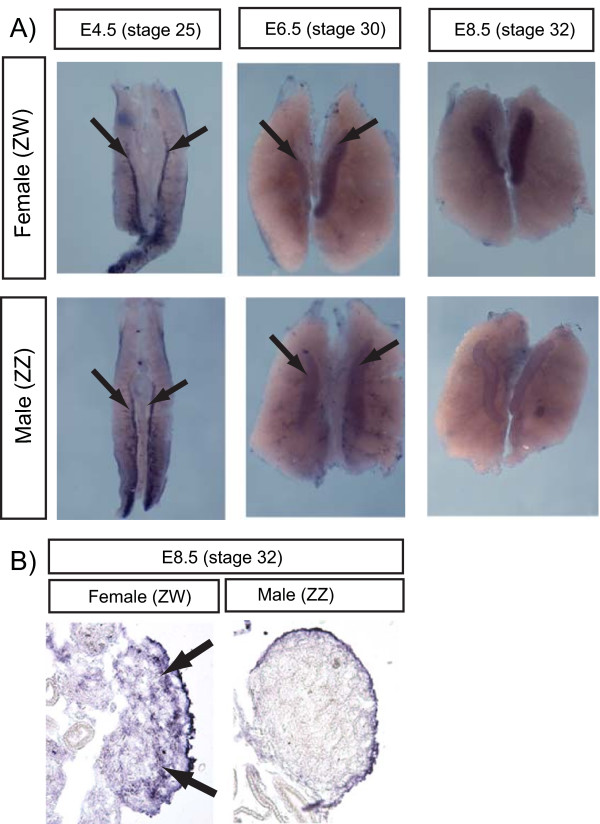
**Gonadal expression of chicken *WNT4*, assessed by *in situ *hybridization**. A) *cWNT4 *is expressed in the gonads of both sexes at E4.5 (prior to sexual differentiation) (arrows). Expression is similar between the sexes at E6.5 (near the beginning of sexual differentiation), but is down-regulated in males (ZZ) by E8.5. B) Transverse sections of over-stained E8.5 whole mounts showing *cWNT4 *expression in the outer region of the female gonad. Weak staining in the male gonad represents background.

The *RSPO1 *expression profile in embryonic chicken gonads was compared with that of *WNT4*, which also signals through β-catenin and is known to be important for proper ovary development in mammals. As assessed by whole mount *in situ *hybridisation, *cWNT4 *was expressed in the gonads of both sexes from E4.5, prior to gonadal sex differentiation. Expression became sexually dimorphic during gonadal sex differentiation (E6.5 – 8.5), being up-regulated in females and down-regulated in males. Little expression was observed in the adjoining mesonephros (Fig. 4A). In sections of over-stained tissues, *WNT4 *mRNA was localised towards the outer (cortical) region of the developing ovary (Fig. 4B).

### RSPO1 expression in a turtle with temperature dependent sex determination

**Figure 5:**
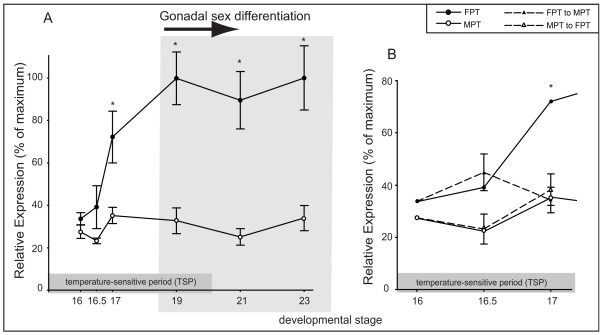
**Expression of *RSPO1 *mRNA in the gonads of turtle embryos (*Trachemys scripta*) during the temperature-sensitive period**. Expression levels were examined by quantitative real-time PCR in three samples per sex/stage. Mean +/- SEM. ● = female; ○ = male. A) Time course of *T. scripta Rspo1 *expression, showing higher expression in developing ovaries from stage 17 (p = 0.0318). B) Turtle *Rspo1 *expression following temperature shifts. Expression data at MPT and FPT at three early stages during the temperature-sensitive period is expanded and compared to the response of gene expression to a shift in temperature. Embryos were shifted at stage 16 from either MPT to FPT (MPT → FPT) or FPT to MPT (FPT → MPT) and gonads dissected for analysis at two subsequent timepoints, stage 16.5 and stage 17. Following a FPT → MPT shift, expression is significantly decreased within one stage from female-typical levels (p = 0.0253) and is not different from male-typical levels (p = 1.0000). Following a MPT → FPT shift, expression has not yet changed from MPT-typical levels. Asterisks indicate statistically significant difference in expression between sex within stage at the p = 0.05 significance level, after correction for multiple pair-wise comparison tests by Tukey's HSD.

In the red-eared slider turtle (*Trachemys scripta*), morphological sex chromosomes are absent and egg incubation temperature determines sex. Incubation at 31°C produces 100% female hatchlings, while incubation at 26°C produces 100% male hatchlings. The thermosensitive period (TSP), when temperature influences sex, spans the middle third of incubation, from embryonic stages 14 – 19/20. Turtle *RSPO1 *expression became elevated at female-producing temperatures (FPT, 31°C), but remained low at male-producing temperatures (MPT, 26°C) (Fig. 5). Up-regulation of *RSPO1 *at the FPT occurred during the temperature-sensitive period (TSP), when temperature directs gonadal sex differentiation into testes or ovaries. Expression became statistically higher in bipotential gonads developing at FPT at stage 17 (p = 0.0318, Fig. 5A) and remained high through gonadal differentiation (stages 19, 21 and 23, p < 0.0001, Fig. 5A). Gonadal *RSPO1 *expression levels changed in response to shifting embryos between temperatures at developmental stage 16, indicating temperature-responsive gene expression. Following a shift from the FPT to MPT, expression levels one stage later were down-regulated, and were not significantly different than male-typical levels (stage 17, p = 0.0253, Fig. 5B). However, gonadal *RSPO1 *levels stayed low in embryos shifted from MPT to FPT (Fig. 5B).

### Spatial expression of RSPO1 protein in developing ovaries

**Figure 6:**
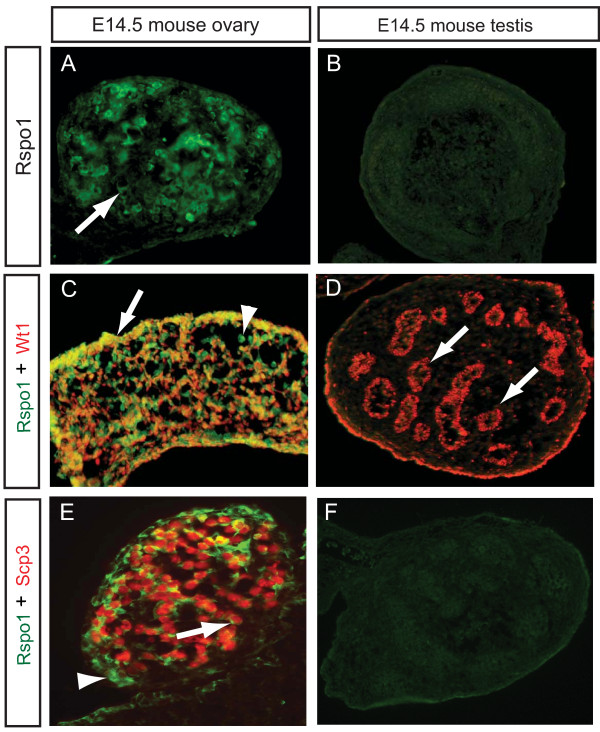
**Immunofluorescent localization of Rspo1 protein in E14.5 embryonic mouse gonads**. A) Rspo1 immunofluorescence in a female gonad. Expression is detectable throughout the gonad, in cell cytoplasm and at the cell surface (e.g.; arrow). Transverse section. B) Background staining in male gonad. Transverse section. C) Double staining for Rspo1 (green) and Wt1 (red) in a female gonad. Most cells that are Rspo1^+ ^and also Wt1^+ ^(e.g., arrow). Some cells stain for Rspo1 but not Wt1 (e.g;. arrowhead). Transverse section. D) Wt1 (red) but not Rspo1 expression in the male gonad (arrows show Wt1 protein in the seminiferous cords). E) Double-staining for Rspo1 (green) and the meiotic germ cell marker, Scp3 (red), showing Rspo1 protein at the germ cell surface (arrow), and also in Scp3^- ^somatic cells (arrowhead). Transverse section. F) Double-staining for Rspo1 (green) and Scp3 (red) shows no expression in the male.

**Figure 7:**
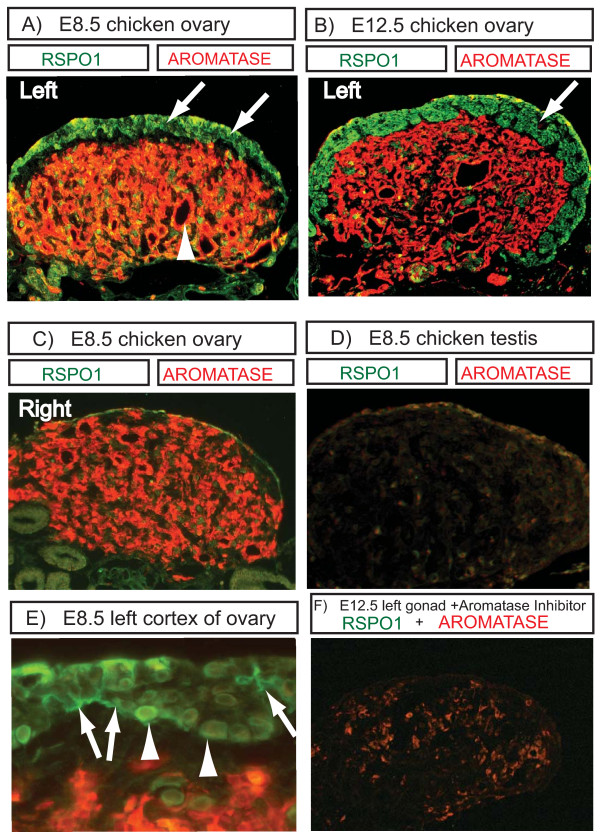
**Immunofluorescent localization of RSPO1 protein in E8.5 and E12.5 embryonic chicken gonads (ten micron transverse sections)**. A) E8.5 left ovary. Double-staining for aromatase (red; medulla) and RSPO1 (green; cortex). B) E12. 5 left ovary. Double-staining for aromatase (red; medulla) and RSPO1 (green; cortex). C) E8.5 right female gonad, showing aromatase expression in the medulla, but no RSPO1 expression due to the reduced cortex. D) E8.5 testis, showing no expression of aromatase or RSPO1. E) High magnification view of the left ovarian cortex, showing RSPO1 expression (green) in the extracellular matrix and at the cell surface (e.g., arrows) and in cell cytoplasm (e.g., arrowhead). F) Loss of RSPO1 protein (green) in an E12.5 female left gonad treated with aromatase inhibitor at E3.5. Aromatase protein is weakly expressed (red), due to fadrozole inhibition, and the cortex is absent.

Immunofluorescence was used to examine the localisation of RSPO1 protein in embryonic mouse and chicken gonads. In E14.5 mouse gonads, Rspo1 protein was detected in the ovary, but not in the testis (Fig 6). In the female, Rspo1 protein was detectable throughout the gonad. The protein was localised in the cytoplasm and cell membrane (Fig. 6A). In females, Rspo1 protein generally co-localised with Wt1, a somatic cell marker, although some Rspo1^+ ^cells were not Wt1^+ ^(Fig. 6C). In E14.5 male gonads, only background staining was detected with the goat anti-mouse antibody used here (Fig 6B). Double staining with the (meiotic) germ cell marker, Scp3, indicated Rspo1 localisation in germ as well as somatic cells of the developing ovary (Fig. 6E), but not in males (Fig 6F). In the chicken embryo, gonads were immunostained at E8.5 (during the period of gonadal sex differentiation) and at E12.5 (when gonadal differentiation is advanced and just prior to the onset of meiosis in females). RSPO1 protein was detected in the outer cortical zone of the developing left ovary. This is the site of germ cell meiosis, and the differentiation of somatic supporting cells into primordial follicles. The underlying medulla, which expresses aromatase protein and synthesises estrogens, was negative for RSPO1 (Fig. 7A and 7B). No protein was detectable in the regressing right female gonad (Fig 7C), consistent with a lack of cortical development. No expression was detectable in the testis (Fig 7D). High power images of RSPO1 protein expression showed cytoplasmic and cell surface localisation among cortical cells of the left female gonad, and potentially some localisation in the extracellular matrix (Fig. 7E). Widespread expression of RSPO1 in the left cortex suggests protein localisation in both somatic and germ cells, as observed in mouse. In gonads taken from female (ZW) embryos treated with aromatase inhibitor, aromatase enzyme expression was significantly reduced and RSPO1 protein could not be detected (Fig 7F). In negative control sections lacking primary antibody, specific staining was abolished (not shown).

## Discussion

These results show that *RSPO1 *has a conserved female-specific expression profile in vertebrate embryos. In all three species, *RSPO1 *mRNA expression becomes elevated in female gonads at the time of somatic cell organization around the germ cells and the onset of germ cell meiosis [24-26]. In the mouse and chicken, RSPO1 expression at this time is predominantly or exclusively localised in the somatic cells. Altogether, these observations implicate RSPO1 in a conserved pathway leading to folliculogenesis and germ cell development. The mouse expression data are in agreement with previous studies showing ovary-specific *Rspo1 *mRNA expression in mammalian embryos [7,14-16,27], and with the impaired follicle development reported in *Rspo1 *null mutant mice [15,16]. Immunofluorescence of Rspo1 protein confirmed a somatic localisation in developing mouse and chicken ovaries, in the cell membrane and around somatic and germ cells. This is consistent with a role of Rspo1 as a secreted signalling molecule [17,19], suggesting that RSPO1 secreted from somatic (pre-follicular) cells has a role in both somatic and germ cell development. This is in agreement with a recent study of embryonic goat gonads, showing somatic and germ cell surface localisation of RSPO1 in the cortical region of the developing ovary [27].

Mammalian RSPO1 has a predicted signal sequence at its N-terminus, and the protein has been reported to be present in the endoplasmic reticulum and Golgi apparatus of transfected 293T cells (human embryonic kidney cells) [19]. Furthermore, all mammalian RSPOs have a thrombospondin motif and they have been shown to bind heparin sulfate proteogylcans on the cell surface and in the extracellular matrix [19,28]. However, a potential nuclear localisation signal has also been identified at the carboxy terminus of mouse Rspo1 and transfected COS7 cells show localisation in the nucleus [17]. Similarly, goat RSPO1 protein translated from a second putative ATG also localises to the nucleus of transfected COS7 cells [27]. The potential role of nuclear localised RSPO1 is unknown, although in transfected cells it appears to associate with nucleoli. However, Western blots show that a number of different RSPO1 isoforms may be produced in transfected cells, some processed for the nucleus and others for the cytoplasm/secretion [17]. In our study, no nuclear localisation of endogenous Rspo1 protein was detected. The commercial antibody used here was raised against the amino terminus of recombinant mouse Rspo1 (amino acids 21–209). It is possible that the antibody fails to detect any nuclear localised RSPO1, if a 5' splice variant lacks amino acids 21–209. Further studies are required to clarify if an endogenous isoform lacking the N terminal signal sequence can localise to the nucleus.

Given that it engages the effector pathway of Wnt signalling, β-catenin, in other systems, it is expected that Rspo1 plays a role in ovarian development by influencing β-catenin. β-catenin has two potential roles in the cell; transactivation of target genes and the formation of adherent-type junctions [29], either or both of which may be important for ovarian differentiation. Rspo1 may act together with Wnt4 in the developing ovary. The spatial and temporal expression profile of *Rspo1 *is similar to that of *Wnt4 *in mouse and chicken; *Wnt4 *is up-regulated in the gonadal somatic cell lineage of presumptive females in both species (Fig 4) [7,8,30]. *Wnt4 *expression is de-regulated in the *Rpso1*^-/- ^mice [15]. In the red-eared slider turtle, *WNT4 *mRNA is expressed at similar levels during the TSP in the gonads of embryos developing at both MPT and FPT, and only becomes dimorphic during later ovarian differentiation [31]. In the mouse embryo, transgenic and knock-out studies have shown that Wnt4 plays a role in ovarian development, by repressing male-typical steroidogenesis and vascular development [8,10]. Furthermore, Wnt4 functions to antagonise the testis-promoting effects of Fgf9 [32]. Some *in vitro *and *in vivo *studies have suggested that, in embryonic ovaries, Wnt4 may function in a non-canonical fashion to block stabilisation of β-catenin [33] and instead sequester it to the cell membrane [34]. However, most recently, it has been shown using *in vivo *transgenic mouse models that Rspo1 and Wnt4 both activate canonical β-catenin signaling in the embryonic ovary, with Rspo1 acting upstream of Wnt4 [15] (*Rspo1 *expression is not affected in XX *Wnt4 *null mutants). While the *Rspo1 *and *Wnt4 *XX null mutants are both masculinized, neither is fully sex-reversed. It is therefore likely that these two factors operate synergistically to activate the canonical β-catenin signaling involved in mouse ovarian development. In this context, it will be of interest to examine the phenotype of *Rspo1*^-/- ^/Wnt4^-/- ^compound mutants, in which complete XX female-to-male sex reversal might occur.

The immunofluorescent studies reported here for mouse and chicken indicate that Rspo1 protein also localises at or in female germ cells (oogonia). However, the qRT-PCR data indicate that transcript expression is low in fetal germ cells, at least in mouse (Fig. 2). The immunofluorescent results may therefore reflect the localisation of secreted Rspo1. Rspo1 is synthesised primarily in somatic cells, but is secreted and becomes localised at the cell membrane and within both somatic and germ cells. Localisation of RSPO1 protein at the cell membrane of both somatic and germ cells was also recently reported in embryonic goat ovaries [27]. This implies that it has a function in both cell types during ovarian development, that is, during the organization of primordial follicles. These observations are consistent with the recent description of the XX Rspo1^-/- ^mice, which are subfertile, with a decline in the number of oocytes and impaired follicle formation [15,16]. This indicates that Rspo1 signaling plays a role in germ cell development and differentiation.

In the chicken embryo, morphological differentiation of the gonads begins at embryonic day 6 – 6.5 (stages 29–30; [35]), when *SOX9 *expression begins in ZZ (male) gonads and *Aromatase *expression begins in ZW (female) gonads [36]. At this time, chicken *RSPO1 *expression was sexually dimorphic, showing higher expression in putative ovaries (Fig. 3). In fact, female-enriched *RSPO1 *expression was detectable as early as E4.5, which precedes Aromatase activation (E6.0). However, the expression seen at E4.5 represents both gonad and mesonephric tissue, as the two tissues could not be easily separated. Therefore, expression in the mesonephros cannot be ruled out. By E6.5, however, gonad-specific *RSPO1 *expression was higher in female gonads compared to males. The *RSPO1 *gene is autosomal in chicken and is therefore not the master sex determinant in birds, which must lie on a sex chromosome/s (Z or W). However, its early sexually dimorphic expression profile in the gonad indicates that *RSPO1 *is upstream in the avian sex-determining pathway, as inferred from the mouse and human studies. The exact onset of gonadal *RSPO1 *expression in the chicken embryo is unclear. Since *WNT4 *is female up-regulated in the embryonic chicken, as in mouse, it is likely that RSPO1 regulates and interacts with WNT4 in birds as in mammals. This molecular aspect of ovarian differentiation may therefore be conserved between birds and mammals.

Chicken RSPO1 protein was localised within the outer cortical zone of the developing left ovary (absent in the right gonad, which regresses during development). The real time PCR analysis showed low levels of *RSPO1 *mRNA at E8.5 (Fig. 3), although strong protein expression was nevertheless detected in the cortex (Fig. 7). This may reflect the small proportion of the gonad that comprises cortex at E8.5, resulting in low *RSPO1 *mRNA levels when expressed relative to whole gonadal mRNA (i.e., the *HPRT *normaliser used here). Thus, proliferation of the left cortex over development correlated with increased *RSPO1 *mRNA levels (Fig. 3). Widespread protein localisation in the cortex of the female chicken embryo suggests both somatic and germ cell expression. Cortical RSPO1 expression may be stimulated by estrogen, as indicated by the aromatase inhibitor studies. Aromatase enzyme is expressed early in avian gonadal sex differentiation, producing estrogen female-specifically from E6.0. In chicken embryos treated with the aromatase enzyme inhibitor, fadrozole, the left ovarian cortex did not proliferate and *RSPO1 *mRNA expression declined (Fig 3). Furthermore, RSPO1 expression could not be detected by immunofluorescence in female gonads after aromatase inhibition (Figure 7). Previous studies have also shown that the left ovarian cortex does not proliferate in chicken embryos treated with aromatase inhibitor, indicating that its development is estrogen dependent [37]. The decline of *RSPO1 *expression after aromatase inhibition may therefore reflect a lack of cortical (pre-follicular) cell proliferation. In the chicken embryo, the transcription factor, *FOXL2*, is expressed female-specifically just prior to aromatase [38,39], and several lines of evidence suggest that FOXL2 activates the *Aromatase *gene during avian (and mammalian) ovarian development [40-42]. Chicken *FOXL2 *and *Aromatase *are both expressed in the gonadal medulla from E5 – 6, while RSPO1 is expressed in the cortex. Activation of *RSPO1 *expression may therefore be independent of the FOXL2-aromatase pathway (as indicated in goat embryos lacking *FOXL2*; [27]) or *RSPO1 *may lie downstream of aromatase in the avian ovarian pathway. However, the aromatase inhibition experiment indicates that estrogen synthesis is required to maintain *RSPO1 *expression, possibly by maintaining the cortical pre-follicular cell population (Note that expression of *FOXL2 *is also retarded in female embryos when aromatase is inhibited, even though FOXL2 expression begins prior to aromatase, implying an estrogen feedback maintenance mechanism; [38]).

*RSPO1 *expression was also sexually dimorphic in the gonads of the turtle, *T. scripta*, a reptile with temperature-dependent sex determination (TSD) [25]. Expression was significantly higher in turtle embryos incubated at the female-producing temperature (31°C), and expression declined in embryos shifted from the female- to male-producing temperature (Fig. 5). Sexually dimorphic expression of *RSPO1 *expression occurred during the temperature-sensitive period (TSP), when bipotential gonads are sensitive to the action of temperature. The temperature-sensitive molecule in reptiles with TSD remains unknown, but genes up-regulated during the critical TSP are likely to be key candidates for early mediators of gonadal development. In *T. scripta*, the TSP spans stages 14 – 19/20, and sexual differentiation of the gonads begins near the end of this period [25]. *RSPO1 *mRNA was more highly expressed in gonads incubated at FPT from stage 17, during the middle of the TSP and before the onset of gonadal sex differentiation. *RSPO1 *may therefore lie upstream in the genetic cascade leading to female development in species with TSD, as inferred for birds and mammals. The temperature shift experiments indicate that *RSPO1 *expression responds to temperature. However, lack of up-regulation in male embryos shifted to the female temperature may indicate that *RSPO1 *up-regulation has not yet occurred within one stage post-shift. Future studies extending analysis to later stages would clarify this point. Factors such as the candidate male determinant *DMRT1 *are male-specific from an early stage during the TSP in *T. scripta *[43,44], and these factors may pre-empt RSPO1.

As in chicken, *FOXL2 *and *aromatase *are up-regulated at the female temperature in *T. scripta *(from stage 18 and stage 19, respectively) [31,45,46]. Therefore, similar to the case in chicken, FOXL2/aromatase may play a role in formation of the ovarian medulla, while RSPO1 may be more important for differentiation that occurs in the outer cortex, namely, primordial follicle development. In egg-laying vertebrates such as birds and reptiles, estrogen synthesised by aromatase is important for stimulating development of the outer cortex of the gonad. It is therefore possible that *RSPO1 *expression is maintained by estrogen during ovarian development in oviparous species, as suggested by the aromatase inhibitor experiments in the chicken [37,47,48]. This may be a direct effect of estrogen on *RSPO1 *expression, or, as discussed above, an indirect effect via maintenance of the pre-follicular cells.

## Conclusion

RSPO1 has a conserved, female-specific expression profile that implies an important role in vertebrate ovarian development. The gene is up-regulated at the earliest stages of ovarian sex differentiation in animals with different sex-determining mechanisms (XY male heterogamety in mammals, ZW female heterogamety in birds, and TSD in a reptile). *RSPO1 *mRNA expression is enriched in ovarian somatic cells, while the protein localises to the cell surface and cytoplasm of both somatic and germ cells. Taken together, the data indicate that RSPO1 has a role in primordial follicle development. Further studies are required to determine how *RSPO1 *expression is regulated in the different groups, and to elucidate its mechanisms of action, likely to be mediated by interaction with Wnt signaling and stabilisation of β-catenin.

## Methods

### Embryos

Mouse strains were either outbred CD1 or, for isolating germ versus somatic cells, OG2 male × CD1 female matings. OG2 mice carry a GFP transgene driven by the *Oct4 *promoter (germ cell specific) [49]. This allowed the isolation, by FACS, of GFP+ germ cells versus GFP- somatic cells. Mouse embryos were collected from embryonic day (E) 12.5 – 16.5. Embryonic day 0.5 was recorded as the morning of vaginal plug detection following overnight matings. From E12.5, the sex of embryonic mouse gonads can be determined by the presence/absence of organising seminiferous cords. Chicken (*Gallus gallus domesticus*) embryos were obtained form a local supplier and incubated under humid conditions at 37.8°C. Embryos were harvested at various days throughout development and staged according to the morphological criteria of Hamburger and Hamilton [35]. Paired gonads were dissected away from the adjoining mesonephric kidneys and tissues prepared for RNA extraction or immunofluorescence (see below). (Except for E4.5, stage 25, where gonads + mesonephros were taken). Tissues were harvested at embryonic day (E) 4.5 (stage 25), E6.5 (stage 31), E8.5 (stage 33), 10.5 (stage 36) and 12.5 (stage 38). In the chicken, gonads are undifferentiated at E4.5, and morphological differentiation begins at E6.0 (stages 29) [50]. For sexing, a small piece of limb tissue was digested in PCR compatible buffer containing proteinase K (200 μg/ml at 50°C for at least 30 minutes), followed by rapid PCR amplification of the sex-linked, female-specific *Xho*1 sequence. Amplification of 18S ribosomal RNA genomic sequence was used as the internal control in a duplex reaction, according to the method of Clinton *et al*. [51]. For aromatase enzyme inhibition, eggs were treated with a single injection of fadrozole, a specific non-steroidal aromatase inhibitor (Novartis, Basel, Switzerland). The fadrozole was dissolved in PBS at a concentration of 10 mg/mL and 0.1 mL (1 mg) was injected into the pointed end of each egg. Controls were injected with 0.1 mL of PBS alone. Holes were sealed with tape and incubation allowed to proceed until E8.5 or E12.5.

Freshly laid red-eared slider (*Trachemys scripta*) eggs purchased from Clark Turtle Farms (Hammond, LA) were maintained as previously described [25,52], in accordance with humane animal practices under IACUC protocol #03102301. Briefly, viable eggs were randomised in trays of moistened vermiculite and placed in incubators (Precision, Chicago, IL) at 26.0°C (a 100% male-producing temperature, MPT) or 31.0°C (a 100% female-producing temperature, FPT). Incubator temperatures were monitored daily with HOBO data loggers (Onset Computer Corp., Bourne, MA) and verified with calibrated thermometers. For temperature shift experiments, multiple trays of 30 eggs/tray were shifted at developmental stage 16 from incubators held at 26.0°C to 31.0°C and vice versa. Progression of development was monitored by staging external morphological characteristics of a sampling of individuals according to Greenbaum's staging series [53]. The temperature-sensitive period (TSP) in the slider turtle spans Greenbaum's stage 14 through stage 19 at a FPT and through stage 20 at a MPT [25]. Shifting eggs during the temperature-sensitive period (TSP) from one end of the temperature spectrum to the other (i.e., from 26°C to 31°C or vice versa) redirects gonadal development, resulting in 100% sex-reversal. Gonadal differentiation begins at stage 18/19 and lasts through hatching at stage 26 [25].

### Mouse *Rspo1 *expression analysis

Gonads from OG2 mouse embryos (expressing GFP in the germ cells) were harvested at E12.5, E13.5, E14.5 and E15.5. Pooled gonads were dissociated in 0.01% trypsin/PBS, filtered, resuspended in PBS and sorted by FACS. This resulted in GFP positive and GFP negative fractions, which represented germ and somatic cells, respectively [23]. RNA was extracted from each fraction, treated with DNase and reverse-transcribed using Superscript III (InVitrogen). Quantitative real time PCR was carried out, using the Universal Probe Library (UPL) system and Faststart Taqman Probe master mix with ROX(Roche). Real time PCR was performed on an ABI 7900 HT instrument. All samples were run in triplicate and normalised against the stably expressed "house-keeper" *Succinate dehydrogenase subunit A flavoprotein *(*Sdha*). The comparative C_T _method (ΔΔC_T_) was used to analyse the data. Rt- and water controls showed no amplification. The primers used for *mRspo1 *amplification were *mRspo1. For*: 5'-AAGGAGTGGAACCTTCTGGAG-3'; *mRspo1.Rev*: 5'-CCAATCTGCCATCCATCTGT-3'. The primers used for *Sdha *were *mSdha.For *5'-TACCTGCGTTTCCCCTCATA-3' ; *mSdha.Rev *: 5'-CACAATCTATGAAGTGACTCCTTGTT-3'.

### Isolation of chicken Rspo1 orthologue and quantitative Reverse Transcription-PCR (qRT-PCR)

RNA was extracted from pairs of gonads over development (E12.5–E15.5 for mouse, E4.5 – E12.5 for chicken and stages 16–23 for turtle). For mouse and chicken, tissues were pooled according to sex for RNA extraction, using the Sigma RNA Elute miniprep kit. Approximately five pairs of gonads were used for each sex at each stage. Total RNA was subjected to DNase treatment to remove contaminating genomic DNA using the Ambion DNA-free kit. For each sample, 400–1000 ng DNased RNA was reverse transcribed using Superscript III™ (InVitrogen) and a mixture of oligo-dT and random hexamers as primers. One μL of each RT reaction was used for each real time PCR reaction. BLAST analysis of the chicken genome identified an orthologue that showed homology with human *R-spondin1 *at the nucleotide level. As for the mouse expression study, primers spanning *cRSPO1 *exon-exon boundaries were designed and used together with the Universal Probe Library (UPL) system and Faststart Probe Master mix. The primers for chicken *RSPO1 *were *cRSPO1. For*: 5'-GGAACGATATCCGGCAAA-3'; *cRSPO1.Rev*: 5'-CTCACAGTTCTCGATTTTGCAT-3'. Samples were run in triplicate and experiments performed at least twice. All samples were normalised against *cHPRT *using the comparative C_T _method (ΔΔC_T_). *HPRT*, which is autosomal in chicken, was found to be a stably expressed during chicken gonadal development. The *HPRT *primers were *cHPRT.For*: 5'-GTGATTGGCGATGATGAACA-3'; *cHRPT.Rev*: 5'-CACGTGCCAGTCTCTCTGTC-3'. For all qRT-PCR data presented here, no template controls or RT- samples exhibited no amplification. In addition, each amplification set was performed with standard curves to confirm primer/probe efficiency of greater than 80%).

### *In situ *hybridization for chicken *WNT4 *expression

A 520 bp chicken *WNT4 *fragment was amplified from mixed sex gonadal cDNA, using the primer pair *cWNT4/For*: 5'-TCTGGCTGCTCCGATAACATT-3' and *cWNT4/Rev*: 5'-ACCTTTCCACCACCTCCACTT-3'. It was cloned into pGEM T Easy vector (Promega), sequenced to confirm identity and used as template for riboprobes synthesis. *In situ *hybridization was performed as described previously [54]. Briefly, urogenital systems were dissected from embryos at E4.5, 6.5 and 8.5. All samples were sexed by PCR as described above. Tissues were fixed overnight at 4°C in 4% paraformaldehyde and processed for *in situ *hybridization with a digoxigenin (DIG)- labeled antisense riboprobe. For negative controls, a DIG-labelled sense riboprobes was generated. Alkaline phosphatase-conjugated Anti-DIG antibodies were used, and the chromagen was BCIP/NBT.

### Isolation of turtle RSPO1 orthologue and quantitative Reverse Transcription-PCR (qRT-PCR)

To isolate the turtle *RSPO1 *orthologue, total RNA was first extracted from pooled adrenal-kidney-gonad (AKG) complexes from each sex at a variety of stages and reverse-transcribed using oligo(dT) primers with Superscript III reverse transcriptase (Invitrogen, Carlsbad, CA). Degenerate primers for *RSPO1 *were designed based on published mouse, human and chicken sequences and were: *forward*: 5'-AAG CTG TTC ATC CTG CTG GAR MGN AAY GA-3' and *reverse*: 5'-CCT TCC TCC TCT TCT GGC CYT CNG GRC A-3'. The amplified fragment was ligated into pCRII-TOPO vector according to the manufacturer's protocol (Invitrogen, Carlsbad, CA) and sequenced using M13F and M13R primers. The turtle homolog *RSPO1 *sequence was submitted to GenBank (accession number pending). Full length turtle *RSPO1 *sequence was cloned using the Smart RACE kit (Clontech, Mountain View, CA) based on this initial sequence, via a nested strategy. Primers were as follows: 3' RACE outer: CCT GCT GA GAG GAA CGA CAT CC, 3' RACE inner: ATC TGC CTG CCC TCC TGT CCG C, 5' RACE outer: TGT CTG GAT TGC GAA CAC CGA AG, 5' RACE inner: CGG ATG TCG TTC CTC TCC AGC AG.

Quantitative RT-PCR was performed as previously described [44]. Briefly, pairs of gonads were dissected (isolated from adjacent adrenal-kidney-mesonephric tissues) from 12 to 20 turtle embryos per independent sample (n = 3 at each sex/stage), pooled, immediately placed in RNA denaturing solution (Promega), vortexed to dissociate, and stored at -80°C. Total RNA was later extracted using the RNAgents Total RNA Isolation kit (Promega) and treated with DNA-Free DNase I (Ambion, Austin, TX). RNA was reverse-transcribed using the SuperScript First-Strand Synthesis for RT-PCR system (Invitrogen) with both oligo-(dT) and random hexamer primers. Relative gene expression levels were quantified using SYBR Green I dye (Invitrogen, Carlsbad, CA) and an ABI PRISM 7900 HT real-time PCR cycler (ABI SDS 2.2.1 software). Samples were each run in triplicate and the median value was used for analysis. PCR efficiencies were calculated from gene-specific standard curves. Relative transcript abundance was normalized to expression of *protein phosphatase 1 *(*PP1*), a transcript selected previously that is constitutively expressed across both stage and sex [31]. A modified delta CT method that allows for correction of differential gene PCR efficiencies was utilized, where mean normalized expression (MNE) is

MNE = mean [(E_ref_^Ct_ref_)/(E_target_^Ct_target_)]

where E = gene-specific PCR efficiency, Ct = cycle threshold from each independent sample, target = gene of interest, and ref = constitutively expressed reference gene. Data were then expressed as percentage of maximum expression. Primers used to assay gene expression were designed across putative vertebrate exon boundaries using MacVector (MacVector, Inc., Cary, NC). Primer specificity was verified by agarose gel electrophoresis, and for *PP1 *were as previously described [31]. Quantitative PCR primers for *Rspo1 *were:*forward *5'-TCG GTG TTC GCA ATC CAG AC-3', *reverse *5'-TTT GTG CAA AAG TTT CGG CTG-3'

### Immunofluorescence

Urogenital tissues were dissected from mouse and chicken embryos and briefly fixed for 15 minutes in 4% paraformaldehyde/PBS at room temperature. Tissues were then cryo-protected by immersion in 30% sucrose/PBS (overnight at 4°C), infiltrated with OCT embedding compound and frozen on dry ice or isopentane pre-cooled in liquid nitrogen. Ten micron frozen sections were cut and thaw mounted onto Superfrost Plus slides. Sections were treated with 1% Triton X-100/PBS (10 minutes), washed in PBS, and then blocked for 1 hr at room temperature in 2% BSA in PBS. Primary antibodies were diluted in 1% BSA/PBS and added to sections overnight at 4°C. Goat anti-mouse RSPO1 antibody was obtained from R&D Systems and used at a dilution of 1:50. Rabbit anti-WT1 was obtained from Santa Cruz Inc. (1:200), while rabbit anti-SCP3 was obtained from Novus Biologicals (1:100). The rabbit anti-aromatase antibody was raised in house and used at 1:5000. After overnight incubation, sections were washed in PBS and secondary antibodies were added in 1%BSA/PBS for 1 hr at room temperature (AlexFluor 488 donkey anti-goat IgG, 1:1000, and AlexaFluor 594 donkey anti-rabbit IgG, at 1:1500). Sections were then washed three times in PBS and mounted in Fluorosave (Calbiochem.). Images were collected on a compound microscope equipped with fluorescent filters.

## Abbreviations

BSA: Bovine Serum Albumen; Ct: Cycle threshold; DMRT1: Double-sex and Mab-3 Related Transcription factor; E: Embryonic days of development; FGF: Fibroblast Growth Factor; FOXL2: Forkhead box transcription factor 2; FPT: Female-Producing Temperature; HPRT: Hypoxanthine guanine PhosphoRibosylTransferase; MPT: Male-Producing Temperature; RSPO1: R-SPONDIN1; SCP3: Synaptonemal Complex 3; SOX9: SRY-like HMG box 9; SRY: Sex-determining Region of the Y; TSD: Temperature-dependant Sex Determination; TSP: Thermonsensitive Period for sex determination; Wnt4: Wingless related MMTV integration site.

## Authors' contributions

All authors participated in the design and execution of the study. CAS and CMS developed the rationale and experimental methods used. CMS, KNR and JQ carried out the cDNA cloning and expression analysis, while CAS performed the immunofluorescence. KNR, CAS and CMS analysed the results. CAS, CMS, AHS and DC drafted the manuscript. All authors read and approved the final manuscript.
